# National Association of Dental Faculties

**Published:** 1890-09

**Authors:** 


### NATIONAL ASSOCIATION OF DENTAL FACULTIES.

The Seventh Annual Session of the National Association of Dental Faculties was held at Excelsior Springs, Mo., commencing Monday, August 4, 1890.

The following colleges were represented:

Baltimore College of Dental Surgery, M. Whilldin Foster.

Boston Dental College, Wm. Barker.

Chicago College of Dental Surgery, Truman W. Brophy.

Kansas City Dental College, J. D. Patterson.

Missouri Dental College, W. H. Eames.

Ohio College of Dental Surgery, H. A Smith.

Pennsylvania College of Dental Surgery, C. N. Peirce.

University of California, Dental Department, C. L. Goddard.

University of Iowa, Dental Department, A. O. Hunt.

University of Michigan, Dental Department, J. Taft.

University of Pennsylvania, Dental Department, James Truman.

Vanderbilt University, Dental Department, D. R. Stubblefield.

Louisville College of Dentistry, A. Wilkes Smith.

Indiana Dental College, J. R. Clayton.

Dental Department of Southern Medical College, L. D. Carpenter.

Dental Department of University of Tennessee, R. B. Lees.

University of Maryland, Dental Department, John C. Uhler.

Columbian University, Dental Department, H. B. Noble.

On motion, Dr. J. D. Patterson, Kansas City, was elected secretary *pro tem.*

The following resolution, offered by Dr. Hunt, was adopted:

"*Resolved,* That in all colleges of this Association students to be graduated at the expiration of two years after admission must enter the school not later than twenty days after the opening of the regular session following this meeting."

The amendment to the constitution laid over from last year, providing for changing the name of the Association to American Association of Dental Faculties, was lost.

Applications for membership laid over last year, under the rules, were taken up and the following were admitted: Royal College of Dental Surgeons of Ontario; College of Dentistry, Department of Medicine, University of Minnesota (represented by Dr. W. X. Sudduth); American College of Dental Surgery (represented by E. P. Hazen).

The following applications for membership were laid over under the rules: Dental Department of Howard University, Washington, D. C., and College of Dentistry, University of Denver.

The resolution offered by Dr. Patterson and laid over last year under the rules was taken up, amended, and adopted as follows:

"*Resolved,* That after the session of 1890–91 a diploma from a reputable medical college shall entitle its holder to enter the second course in dental colleges in this Association, but he may be excused from attendance upon lectures and examinations upon the following subjects: general anatomy, chemistry, physiology, and materia medica and therapeutics."

Dr. Marshall's amendment to the constitution, providing that in all matters not in conflict with Article V. of the constitution a majority of the colleges belonging to this Association shall constitute a quorum, was taken up and adopted.

The following resolution, offered by Dr. Hunt, was adopted:

"*Resolved,* That we recommend that students take two full courses in studies of a general character, such as anatomy, physiology, chemistry, general principles of surgery, and materia medica and therapeutics, and three courses in those of a special dental character."

Dr. Goddard offered the following resolution, which was adopted:

"*Resolved,* That final examination may be taken at the end of the second year in three general studies."

The following, offered by Dr. Truman last year and laid over under the rules, was adopted:

"*Recommended*, That for a full annual course of lectures the minimum sum of college fees be $100; that diploma fees be omitted, and an examination fee of not less than $25 be substituted therefor and made non-returnable; that a matriculation fee of $5 be charged annually. Special-course fees to be $10 for each branch taken, and $5 matriculation fee."

The following officers were elected for the coming year: L. D. Carpenter, Atlanta, Ga., President; W. H. Eames, St. Louis, Mo., Vice-President; J. D. Patterson, Kansas City, Mo., Secretary; H. A. Smith, Cincinnati, O., Treasurer; J. Taft, Cincinnati, O., Truman W. Brophy, Chicago, and A. O. Hunt, Iowa City, Ia., Executive Committee.

The following committees were appointed: James Truman, Philadelphia; Frank Abbott, New York; and John S. Marshall, Chicago, *ad interim* committee; J. A. Follett, Boston; D. R. Stubblefield, Nashville, Tenn.; A. Wilkes Smith, Richmond, Ky.; C. L. Goddard, San Francisco, committee on schools.

Adjourned to meet on Saturday, August 1, 1891, at 10 o'clock A.M., at the place appointed for the next meeting of the American Dental Association.

---

